# Palisade Cartilage Tympanoplasty, an Alternative Surgical Approach for CSOM

**DOI:** 10.22038/IJORL.2022.60937.3093

**Published:** 2022-07

**Authors:** Shahid Rasool, Shifa Qureshi, Ashima Varshney, Simmi Hassan, Faiza Kokab, Khaja Naseeruddin

**Affiliations:** 1 *Department of Otorhinolaryngology Head and Neck Surgery, Hamdard Institute of Medical Sciences, New Delhi, India.*

**Keywords:** Cartilage Tympanoplasty, Sclerotic Mastoids, Myringoplasty, Type 1 Tympanoplasty

## Abstract

**Introduction:**

The hearing outcome and graft take in patients of CSOM with sclerotic mastoids were studied using the novel technique of palisade cartilage tympanoplasty. Besides, it was compared with tympanoplasty type-1 above and over the cortical mastoidectomy in both groups.

**Materials and Methods:**

Out of 313 patients of CSOM, 125 had sclerotic mastoid and were included in the study. Palisade cartilage group patients were subjected to palisade cartilage tympanoplasty type-1. While as in the Temporalis fascia group patients, type-1 tympanoplasty was done using temporalis fascia as graft material. These procedures were performed in addition to cortical mastoidectomy done in all cases.

**Results:**

Statistically significant (P<0.001) mean postoperative hearing gain was achieved (> 20 dB) in both the groups with a reduction of AB gap to 13.3 & 11.79 dB, respectively. However, the post-surgery hearing outcomes achieved were similar in both groups (P=0.09). The overall graft take rate of 86% was seen in the Palisade cartilage group. The remaining 14% had graft take failure. The primary graft failure rate was 10% (5/50), and the secondary failure rate within six months of follow-up was 4% (2/50). The Temporalis fascia group graft take rate was higher (92%) than the Palisade cartilage group, with only 4 % (3/75) of cases having a primary graft failure rate. However, these findings (92% vs. 86%) were not statistically significant (P=0.2830).

**Conclusions:**

As the hearing outcomes and graft take rates were comparable in the two groups, the present study highlighted the use of palisade cartilage tympanoplasty in patients of CSOM with sclerotic mastoids as an alternative method to tympanoplasty.

## Introduction

Chronic otitis media (COM) is an inflammatory process of the mucoperiosteal lining of the middle ear cleft. Almost 6 % of the Indian population suffer from chronic ear disease (1). Tympanoplasty (TP) is a surgical procedure for removing the middle ear disease in patients with COM to achieve a dry ear and reconstruct the hearing mechanism (2). 

It is the most commonly performed surgery for COM in otology. There are many approaches for tympanoplasty, and so are graft materials used to repair the tympanic membrane (TM). The most commonly used graft material is temporalis fascia. However, it is quite possible to have re-perforation or retraction of the eardrum after reconstruction with temporalis fascia graft, which can change its shape due to uneven shrinkage and thickening, even in the early days of the procedure (3). 

The instability of the temporalis fascia is critical in cases where perforations of the TM are considerable (4) and are associated with Eustachian tube dysfunction. In addition, the presence of sclerotic mastoid in COM has been considered as one of factors affecting the graft take adversely following tympanoplasty (5). 

To over come these issues, many otologists nowadays routinely use cartilage as graft material, while others perform cortical mastoidectomy over and above the tympanoplasty. The cartilage's benefits are that it maintains its firmness and resists resorption and retraction, even in the milieu of continuous eustachian tube dysfunction (6). The cartilage tympanoplasty technique consists of a heterogeneous group of techniques, including the cartilage-perichondrium composite graft, butterfly techniques, diced cartilage, and palisade cartilage tympanoplasty (7-9). 

As the cartilage is thicker than temporalis fascia, its vibrations are mechanically moderated compared to the vibration of the fascia tympanic membrane and thus leads to some impairment in the functional hearing outcome. 

However, it has been observed that vibration characteristics of the cartilage graft are similar to fascia graft when thinned out and used in the form of palisades (10,11). Thus Palisade cartilage repair of the tympanic membrane improves its mobility and lessens the acoustic impedance compared with larger pieces of cartilage (12). Furthermore Palisade cartilage tympanoplasty has been seen to restore the same degree of auditory function as in tympanoplasty using temporalis fascia (13). The objective of the current study was to analyse the hearing outcome and graft take by performing palisade cartilage tympanoplasty in patients of CSOM with sclerotic mastoids as an alternative to tympanoplasty and compare the results of the two different techniques used.

## Materials and Methods

The current study is an experimental comparative prospective study done at the department of ENT- Head and Neck Surgery of Hamdard Institute of medical sciences new delhi, a tertiary care hospital for three years, from June 2018 to June 2021. Three hundred and thirteen chronic suppurative otitis media (CSOM) patients were evaluated for surgical intervention. Complete Ent examination of all patients was done. The hearing assessment was done by tuning fork tests and pure tone audiometry (PTA), preferably within one month before surgery. 

Mastoid imaging was done in all patients to assess for pneumatization, including x-rays and/or CT / MRI as and when indicated. All Patients of CSOM with sclerotic mastoids evident on imaging were included. Patients with cholesteatoma, revision surgery, or in whom ossicular erosion was noted intraoperatively were excluded from the study. Out of 313 patients, only 125 fulfilled the study design and were randomly divided into two groups, A (palisade cartilage) & B (temporalis fascia). 

**Fig 1:**
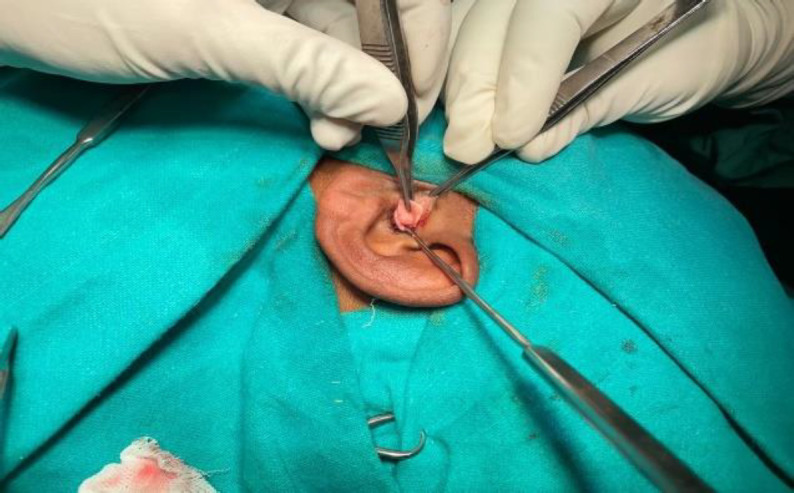
Harvesting of tragal cartilage

**Fig 2:**
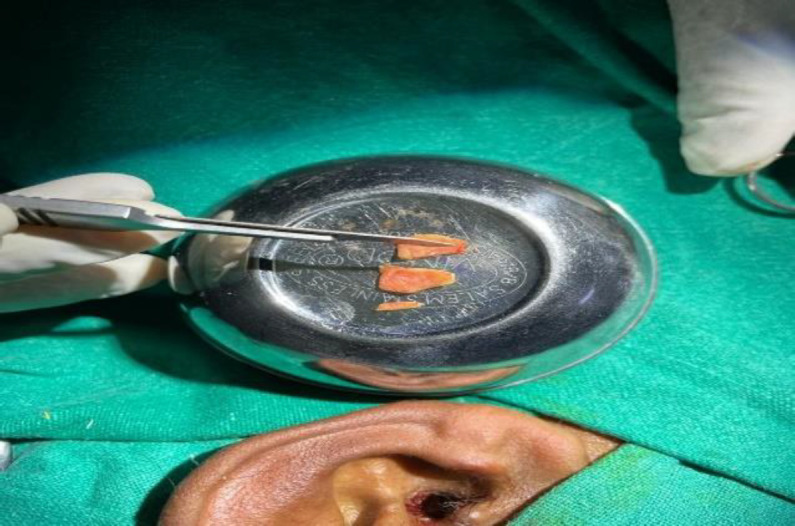
Palisading of tragal cartilage

The palisade cartilage group consisted of 50 patients and the temporalis fascia group of 75 patients. Proper written consent was taken from all patients after explaining the procedure and associated risks. Institutional ethical committee clearance was sought, and approval was given. The first author operated on all cases to reduce surgical bias. In the Temporalis fascia patients, type-1 tympanoplasty was done. While as Palisade cartilage group patients were subjected to palisade cartilage tympanoplasty type 1. In this technique (Fig 1,2), thin & small pieces of cartilages (tragal / conchal) were used as graft material. These pieces were put medial to the fibrous annulus and parallel to the malleus handle and secured with gelfoam. 

These techniques were added to cortical mastoidectomy done in both groups. Which is an institutional policy for all COM patients with sclerotic mastoids. Patients were put on antibiotics and analgesics for one week. After a week, mastoid dressing and canal pack were removed. Follow-up post-surgery was done at two weeks, four weeks, and six months of postoperative period to check for graft take and hearing assessment. Postoperative PTA was done at six months of follow-up in all cases. The hearing outcome was computed by comparing the pre and postoperative pure tone audiometric results. Data were tabulated, and statistical analysis was done by applying the student’s t-test and Chi-Square tests.

## Results

Out of 125 cases of Chronic Suppurative Otitis Media (CSOM) with sclerotic mastoids, 66 were males and 59 were females, with a male-female ratio of 1.1:1. The mean patient age was 27.2 ± 9.965, ranging from 11 to 64 years. Table 1 shows the sex distribution of cases.

**Table 1:** Sex Distribution of Patients

**Groups**	**Male**	**Female**	**Total**
Group A	24	26	50
Group B	42	33	75
TOTAL	66	59	125


Thirty patients had bilateral disease at the time of examination, while the rest 95 had unilateral disease only. Most patients had mild to moderate hearing loss. The mean preoperative hearing losses (Air Bone gap=AB) in the Palisade cartilage group & Temporalis fascia group were 37.0 & 32.75 decibels (dB), respectively. The mean postoperative hearing gain achieved was more than 20 dB in both the groups (23.7 vs. 20.96), with a reduction of AB gap to 13.3 &11.79 dB, respectively. These findings were statistically significant as both the groups had sufficient hearing gain postoperatively (*P*<0.001). However, the post-surgery hearing outcome when compared between the two groups were quite similar (*P*=0.09). The palisade cartilage technique group had an overall graft take rate of 86%. The remaining 14 % had graft uptake failure. The primary graft failure rate was 10% (5/50), and the secondary failure (re-perforation) rate within six months of follow-up was 4% (2/50). The Temporalis fascia group graft take rate was higher (92%) than the Palisade cartilage group, with only 4 % (3/75) of cases having a primary graft failure rate. However, these findings (92% vs. 86%) were not statistically significant (*P*=0.2830), as shown below in Table 2 as surgical outcomes.

**Table 2:** Surgical Outcomes

**Surgical outcomes**	**GROUP A(palisade cartilage )**		**GROUP B (Temporalis Fascia)**	
	(n=50)	Percent %	(n=75)	Percent %
Graft Take	43	86%	69	92%
Graft Failure	5	10%	3	4%
Re-Perforation	2	4%	3	4%
Others	1 (Perichonditis)	2%	1(Mastoid Tenderness)	1.33%

## Discussion

Heermann first described the palisade cartilage technique in 1962. The use of palisade cartilage technique tympanoplasty is indicated in many cases of CSOM with sub/near-total perforations, retraction pockets, middle ear adhesions, and atelectasis. In addition, some Otologists routinely use cartilage in tympanosclerosis, thermal perforations, and residual defects after primary tympanoplasties. 

The use of cartilage for tympanic membrane repair is well described and has reported advantages of long-term graft survival (6,14,15), low recurrence, lower infection rates, and decreased formation of tympanic membrane retraction pockets over time (15-17). 

Furthermore, cartilage offers good resistance to infections and impaired vascularization (18). In addition, the palisade cartilage technique can resist the extreme barometric changes that occur during ascent or descent (19). In the current study, we did not find a statistically significant difference in graft take or hearing outcome in the two groups under comparison. However, graft take results were higher in the Temporalis fascia group compared to the palisade cartilage group (92 % VS 86%). Our results are supported by the studies of Arora N and Passey J. C. et al. (19). They have compared temporalis fascia with palisade cartilage as graft material in type -1 tympanoplasty and found no significant difference statistically in postoperative hearing or graft take between the two groups.

Sohil Vaidya et al. (20) noted similar statistically insignificant findings in their study where they had compared Modified Cartilage Shield tympanoplasty with simple tympanoplasty. However, the better graft take results in the palisade cartilage group in their study as against our findings could be because they used cartilage shield tympanoplasty where both cartilage and temporalis fascia graft is used, unlike our palisade cartilage technique where palisade cartilages are put parallel to handle of malleus without reinforcement by temporalis fascia. Similarly, Jalali et al. (21) also reported no significant difference in postoperative hearing outcomes between cartilage and TF grafting groups in a systematic review of 3606 patients.

Shishegar M et al. (22) found 100% graft acceptance in the palisade cartilage tympanoplasty group compared to the temporalis fascia group (92.5%), although they concluded that their findings were not statistically significant. Their findings are against our findings, as in the current study, a higher rate of graft take in palisade cartilage group (A) was not observed. The possible reason could be the learning curve. The author acknowledged that over time, the difference in the graft take between the two groups narrowed down as the hands-on experience for the palisade cartilage technique improved after the initial few cases. The current study aimed to analyze the hearing outcome and graft take with an alternative palisade cartilage tympanoplasty technique. As the cartilage is thicker than temporalis fascia, its vibrations are mechanically moderated compared to the vibration of the fascia tympanic membrane and thus leads to some impairment in the functional hearing outcome. However, it has been observed that vibration characteristics of the cartilage graft are similar to fascia grafts when thinned out and used in the form of palisades (10,11). 

Besides that, it has been found that Palisade cartilage repair of the tympanic membrane improves its mobility and lessens the acoustic impedance compared to larger pieces of cartilage (12).

## Conclusion

The author concludes that the use of palisade cartilage tympanoplasty in patients of CSOM with sclerotic mastoids can be an alternative to tympanoplasty as the hearing outcomes and graft take rates are comparable in the two groups. The advantage of palisade cartilage tympano- plasty is that without compromising hearing outcomes as mobility of graft is improved by palisading the cartilage graft into pieces, it overcomes the adversaries of mastoid sclerosis-like retraction pockets, middle ear adhesions, atelectasis, etc. 
